# Editorial: Grey matters in the lab: utilizing human brain tissue for basic research, disease modeling and drug development

**DOI:** 10.3389/fnsyn.2026.1910520

**Published:** 2026-07-31

**Authors:** Viktor Szegedi, Danqing Yang, Dirk Feldmeyer, Karri Lamsa

**Affiliations:** 1 Hungarian Centre of Excellence for Molecular Medicine (HCEMM), Szeged, Hungary; 2 Szegedi Tudomanyegyetem, Szeged, Hungary; 3 Julich Research Center, Helmholtz Association of German Research Centres (HZ) Jülich, Jülich, Germany; 4 Rheinisch-Westfalische Technische Hochschule Aachen, Aachen, Germany

**Keywords:** adaptation, brain slice, cell culture, human, neocortex, oscillation, preclinical, synaptic plasticity

What makes the human cortex stand out? For many years, scientists have leaned heavily on animal models, especially rodents, to uncover fundamental principles of brain function. While these studies have provided valuable insights, recent research involving live human cortical tissue has revealed that human neurons and circuits are not just larger versions of those in rodents. In fact, human cortical networks appear to operate under a distinct set of structural, physiological, and metabolic constraints. The contributions in this Research Topic delve into these differences at various levels, from the morphological and electrophysiological properties of single neurons, calcium dynamics in dendrites, excitatory and inhibitory in neuronal microcircuits, to the mechanisms of brain oscillations, and evolutionary changes in specialized neurons.

A key takeaway from these studies is that the human cortex is organized around a delicate balance between information processing, circuit stability and metabolic efficiency.

Several articles in this Research Topic focus on how the excitatory signals in the human neocortex are regulated. For instance, Márton et al. explored the consequences of inhibiting astrocytic glutamate uptake in human cortical tissue. When glutamate transporters were blocked in neocortical pyramidal neurons, they observed slow inward currents as well as tonic currents that were both mediated by NMDA receptors, but these were not identical. Slow inward currents were strongly affected by blocking a specific NMDA receptor type, while tonic currents showed only partial sensitivity. Interestingly, these effects seemed to decrease with age and were largely absent in tissue from older patients. This suggests that ambient glutamate signaling in the human cortex is dynamic and context-dependent, becoming particularly important under pathological conditions when glutamate homeostasis is disrupted.

A recurring theme among the papers in this Research Topic is the vital role of fast-spiking interneurons (FSINs) in synaptic signaling in the human neocortex. These neurons are crucial for regulating the excitatory/inhibitory balance in the cortex and are involved in the timing and synchronization of oscillatory activity and their unique specialization in humans is highlighted in several of the studies of this Research Topic.

For example, Yoon et al. found that when high-frequency electrical stimulation is applied, FSINs are preferentially engaged while excitatory neurons are suppressed. Notably, FSINs maintain AP firing even during extended periods of stimulation, while excitatory neurons and non-FSINs rapidly become inactive. This stimulation also gradually alters the balance of synaptic activity, so that inhibition within the network is enhanced. Such findings have important implications for the treatment strategies of epilepsy and other disorders, indicating that stabilizing the excitatory drive may involve selectively activating inhibitory microcircuits rather than globally suppressing neural activity.

Similarly, Sandle et al. noted that activating group I metabotropic glutamate receptors boosts excitatory synaptic transmission predominantly onto FSINs rather than other interneuron types. While similar effects were seen in rodents, the underlying mechanisms were different, underlining a core message of this Research Topic: human and animal brains may achieve similar circuit functions through different cellular pathways.

The discussion surrounding the specialization of inhibition is deepened by Szegedi et al., who approach FSINs from an evolutionary and energy-efficiency angle. FSINs are among the most energy-demanding neurons in the brain. However, human FSINs exhibit several adaptations that reduce their energy expenditure while preserving rapid signaling capabilities. Increased input resistance, unique organization of ion channels, simpler dendritic structures, and specialized synapses all suggest a system designed not just for speed, but for energy efficiency as well. In a densely packed cortex, minimizing the energy cost of inhibition likely played a significant role in our evolutionary development.

Looking at individual neurons, multiple studies have underscored the sophisticated ways human cortical cells process information. Szöts et al. studied calcium transients induced by action potentials in neocortical layer 2/3 pyramidal neurons, revealing impressive spatial complexity along dendrites. Calcium signals peaked not at the cell body but rather in intermediate dendritic regions, with various dendritic compartments engaging distinct calcium channels. This paints a picture of highly specialized dendritic computation rather than mere passive signal transmission.

Tore et al. expanded on this by reviewing several human-specific features of neurons that may boost information processing. Human pyramidal neurons have lower membrane capacitance, recover faster from synaptic depression, and possess more complex dendritic arrangements than their rodent counterparts. Together, these traits likely enhance the computational bandwidth and flexibility in a neuron. They also influence how human neurons respond to neuromodulators and pharmacological compounds, highlighting the critical need for direct studies using human tissue in translational neuroscience.

At the network level, Yang and Feldmeyer reviewed rhythmic activity in human brain slices and highlighted a central challenge for the field: oscillatory dynamics are profoundly shaped by both biological and methodological factors. Differences in tissue source, slice preparation, recording conditions, and induction strategies can substantially alter the frequency, stability, and spatial organization of network rhythms. Rather than viewing this variability as a drawback, the authors suggest that it reflects the inherent adaptability of human cortical circuits. Oscillations arise from the interplay of excitation, inhibition, neuromodulation, and the overall condition of the tissue, and shifts in these dynamics may provide valuable insights into different neuropsychiatric disorders.

Several contributions also stress that advancing human neuroscience hinges not only on new findings but also requires innovative experimental approaches. Straehle et al. introduced the Freiburg framework, a comprehensive platform that integrates electrophysiology, imaging, molecular analysis, Raman microscopy, and clinical data. A key strength of this framework is its focus on systematically addressing biological variability among human samples. Given the inevitable heterogeneity in human tissue studies, this framework views variability not as noise but as a vital source of biological and clinical knowledge.

Libé-Philippot situate many findings within a broader evolutionary and developmental context. Human neurons arise from extended developmental processes and species-specific molecular changes that shape their excitability, synapse formation, and circuit maturation. These pathways are often affected in different neurodevelopmental disorders, suggesting that some of the features underlying human cognitive specialization may also confer unique vulnerabilities.

Lastly, Eichler et al. delve into synapses located in the supragranular cortical layers, a region significantly expanded in humans. Their review highlights the distinctive structural and functional characteristics of human synapses, as well as the mechanisms of synaptic plasticity that may facilitate long-range integration and complex cortical functions. Many studies in this Research Topic repeatedly identify supragranular layers as critical sites of human specialization.

In summary, the papers in this Research Topic depict the human cortex as a complex system shaped by various competing demands. Human neurons and circuits are not only optimized for computational capacity but also for stability, flexibility, and energy efficiency. Excitation needs careful regulation, inhibition must be quick yet energy-conscious, dendrites need to enable very complex integration, and networks should balance synchrony with adaptability.

Most importantly, these studies reinforce the idea that to truly understand the human brain, we must study human brain tissue directly. While models involving rodents remain invaluable, many mechanisms identified here—including specific synaptic dynamics, adaptations of interneurons, and dendritic signaling characteristics—cannot be completely understood through animal studies alone.

As we gain better access to human tissue, develop advanced analysis techniques, and refine computational methods, neuroscience is evolving toward a more comprehensive understanding of how human cortical circuits function at different levels. The findings collected here mark a significant shift: from viewing the human cortex merely as an extension of animal neuroscience to recognizing it as a unique system with distinct organizing principles.

